# Unusual Association of a Thymic Cyst and Thymolipoma: A Case Report

**DOI:** 10.7759/cureus.105660

**Published:** 2026-03-22

**Authors:** Kholoud Zahouani, Akram Assadiki, Anass Haloui, Nassira Karich, Amal Bennani

**Affiliations:** 1 Department of Pathology, Mohammed VI University Hospital, Faculty of Medicine and Pharmacy of Oujda, Mohammed First University, Oujda, MAR; 2 Department of Thoracic Surgery, Mohammed VI University Hospital, Faculty of Medicine and Pharmacy of Oujda, Mohammed First University, Oujda, MAR; 3 Department of Anatomopathology, Faculty of Medicine and Pharmacy of Oujda, Mohammed First University, Oujda, MAR

**Keywords:** anterior mediastinal mass, anterior mediastinum, benign thymic lesions, differential diagnosis, incidental thymic mass, thymic, thymic cyst, thymic tumors, thymolipoma, total thymectomy

## Abstract

Benign thymic lesions are uncommon and mainly include thymic cysts, thymic hyperplasia, and thymolipoma. Their coexistence is exceptionally rare and may represent a significant diagnostic challenge, particularly because of the potential for confusion with malignant tumors of the anterior mediastinum. We report the case of a 68-year-old woman who presented with a large anterosuperior mediastinal mass that was radiologically suggestive of a thymoma, in the absence of clinical or biological features of myasthenia gravis. Complete surgical excision was performed, and histopathological examination revealed a multilocular thymic cyst arising in a thymolipoma, characterized by multiple cystic spaces lined by a benign epithelium and thymic parenchyma largely replaced by mature adipose tissue containing residual thymic islands with Hassall's corpuscles, without cytological atypia or evidence of invasion. This case highlights the limitations of imaging in the assessment of anterior mediastinal masses and emphasizes the pivotal role of histopathological analysis in distinguishing benign thymic lesions from malignant thymic tumors, thereby avoiding overdiagnosis and unnecessary aggressive treatment, with complete surgical resection providing an excellent prognosis.

## Introduction

Thymic tumors are uncommon neoplasms arising in the anterior mediastinum and account for approximately 20% of all mediastinal tumors. This heterogeneous group includes thymic cysts, as well as other lesions, such as thymomas and thymolipomas [1]. A thymic cyst is an infrequent benign lesion of the thymus that may be either congenital or acquired. It most often presents as an incidental mediastinal mass and is only exceptionally diagnosed in adulthood [2]. Thymolipoma is a benign tumor of the anterior mediastinum composed of a mixture of normal thymic tissue and mature adipocytes, representing only 2%-9% of all thymic neoplasms [3].

Although each of these entities is rare when considered separately, their coexistence in the same patient is exceedingly uncommon, with only a limited number of cases reported in the medical literature, particularly in the absence of associated conditions, such as myasthenia gravis. Therefore, the description of a case combining a thymic cyst and a thymolipoma highlights an exceptionally unusual presentation that contributes to the current understanding of thymic pathology and raises specific diagnostic and therapeutic challenges for clinicians [4].

## Case presentation

We report the case of a 68-year-old woman with a medical history significant for hyperthyroidism and arterial hypertension, who presented with progressive asthenia over a one-month period. Thoracic computed tomography revealed a relatively well-circumscribed soft tissue mass located in the anterosuperior mediastinum, demonstrating isodense attenuation with homogeneous enhancement following contrast administration. Based on its location and imaging characteristics, a thymic tumor, particularly a thymoma, was initially suspected (Video 1). The patient was clinically asymptomatic, with no respiratory or cardiovascular complaints and no evidence of mediastinal compression. There were no clinical or laboratory findings suggestive of myasthenia gravis or other thymus-associated autoimmune disorders. The serum level of acetylcholine receptor antibodies was within the normal range (<0.4 nmol/L), further arguing against an associated myasthenia gravis.

**Video 1:** Mediastinal window of a contrast-enhanced chest CT scan of the patient. Identification of a relatively well-circumscribed soft tissue mass located in the anterosuperior mediastinum, demonstrating isodense attenuation with homogeneous enhancement following contrast administration. The lesion measures 59 × 34 mm, with a craniocaudal extension of 74 mm (yellow arrow) and exhibits close anatomical relationships with the ascending aorta, the aortic arch, the main pulmonary artery trunk, and the right atrial appendage.

**Figure 1:**
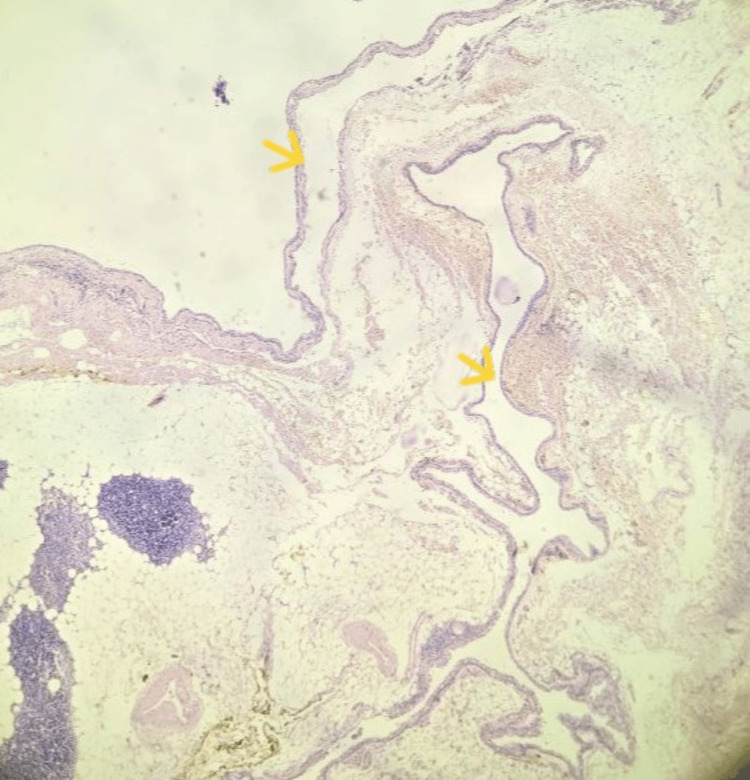
Microscopic image of a surgical excision specimen from the thymic mass showing multiple cystic spaces (H&E, 10x). The cystic cavities are indicated by the yellow arrows.

**Figure 2:**
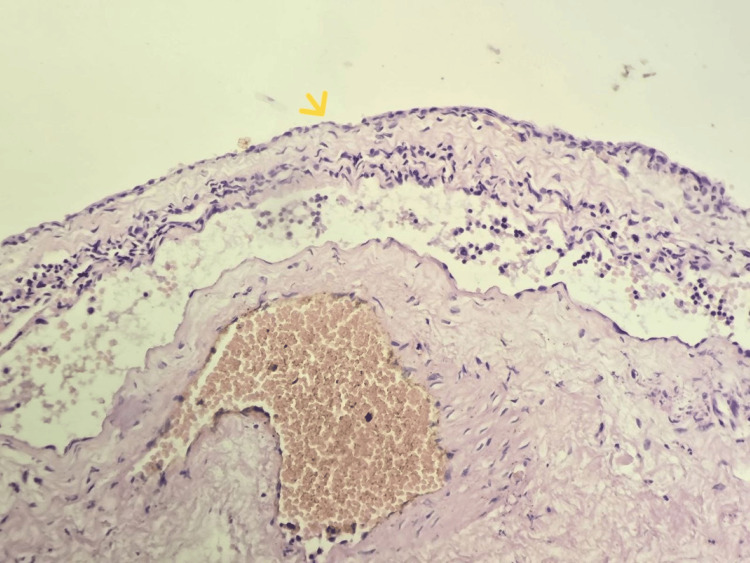
High-power microscopic view of the epithelial lining of the cysts (40x). The cysts are lined by a simple squamous epithelium without cytonuclear atypia (yellow arrow).

**Figure 3:**
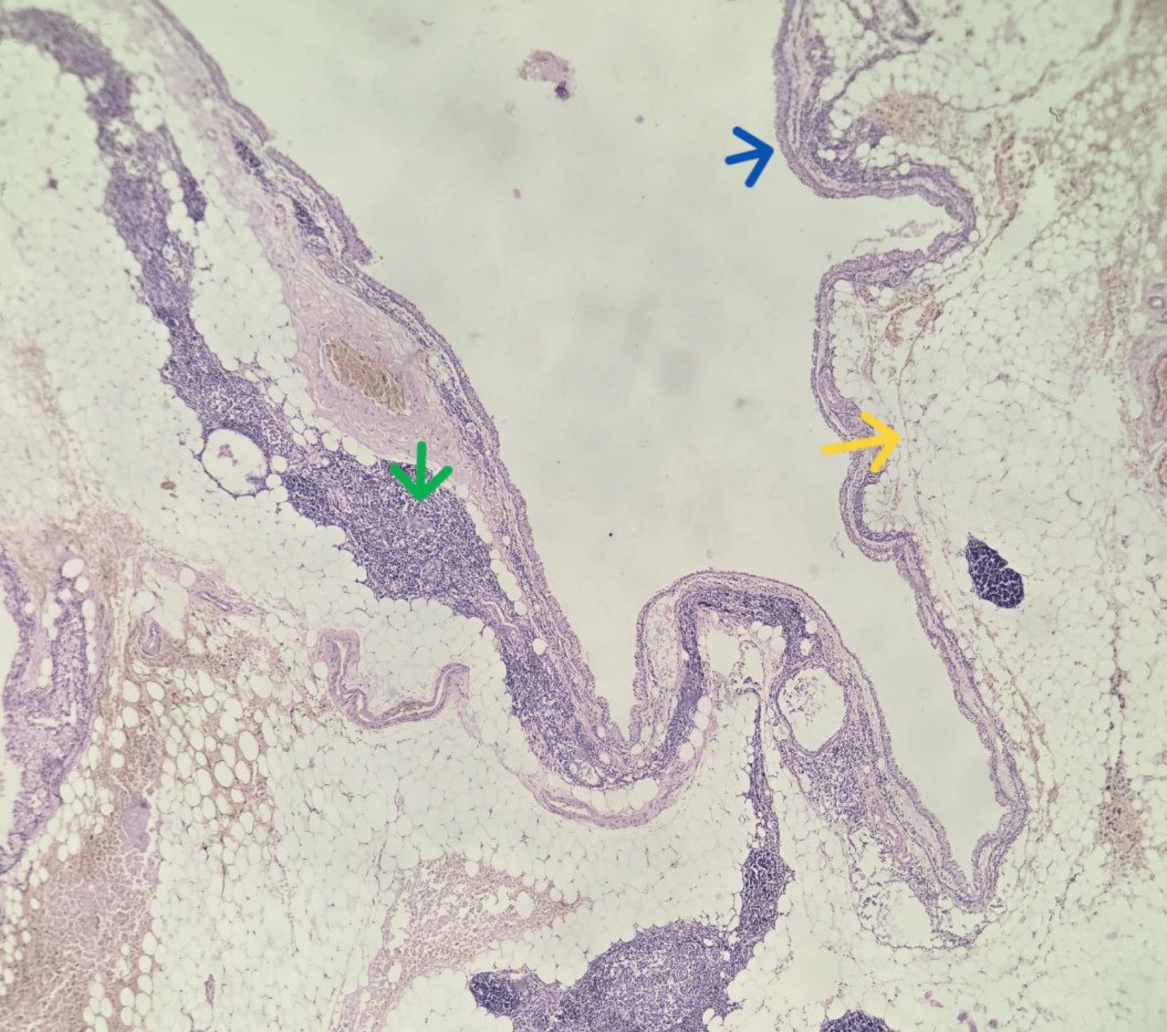
Histological section illustrating the development of a multilocular cyst within a thymolipoma (10x). The thymic parenchyma is largely replaced by mature adipose tissue (yellow arrow), with persistence of a few residual thymic islands (green arrow). The epithelial lining of the cystic cavities is highlighted in blue.

**Figure 4:**
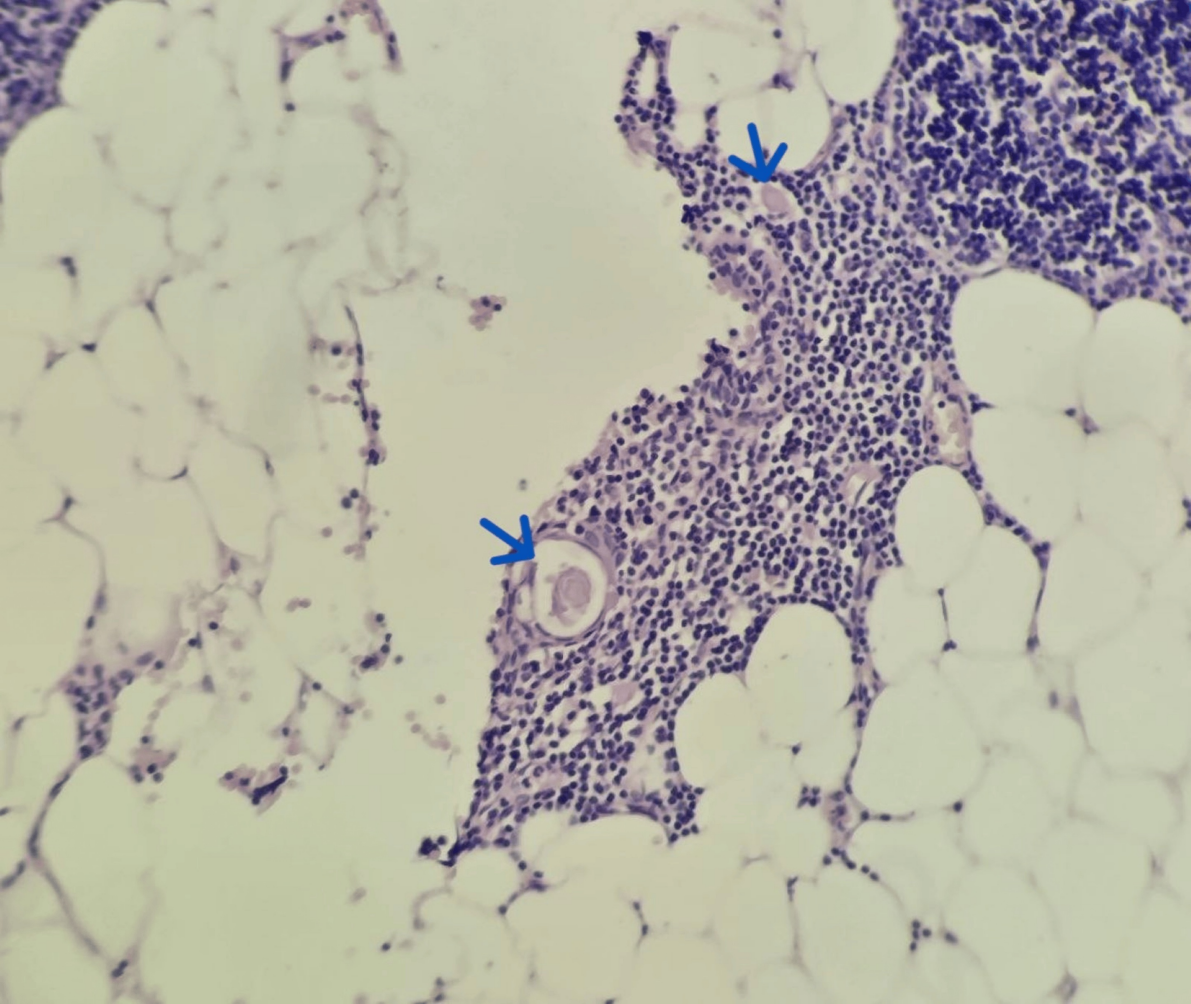
Microscopic image highlighting the presence of Hassall's corpuscles within the thymic tissue (40x). Hassall's corpuscles are indicated by the blue arrow.

Given the size of the lesion and the inability to definitively exclude a malignant thymic neoplasm, the patient underwent complete surgical resection in the Thoracic Surgery Department of Mohammed VI University Hospital in Oujda, Morocco. The surgical specimen consisted of an oriented thymic mass measuring 13 × 5.5 × 0.4 cm and weighing 17.6 g. On cut section, the lesion showed a predominantly fatty appearance involving most of the mass, associated with scattered areas of residual thymic parenchyma with a normal gross appearance. Histopathological examination demonstrated multiple cystic cavities (Figure 1) lined by a simple squamous epithelium, focally discontinuous, without cytological atypia or mitotic activity (Figure 2). The residual thymic parenchyma was largely replaced by abundant mature adipose tissue, within which scattered thymic islands (Figure 3) containing Hassall's corpuscles were identified (Figure 4). The final diagnosis was consistent with a multilocular thymic cyst arising in a thymolipoma.

## Discussion

The association between a thymic cyst and a thymolipoma represents an exceptional condition that has been very rarely described in the medical literature, making its clinical and anatomopathological recognition particularly challenging. To date, only a limited number of isolated cases have been reported, without the identification of a clearly defined nosological entity or a shared pathophysiological mechanism [4].

Thymolipoma is a rare benign tumor of the thymus, composed of a variable admixture of mature adipose tissue and normal thymic tissue. It accounts for approximately 2%-9% of thymic tumors, according to published series. Its slow growth often leads to a delayed diagnosis or incidental detection during imaging studies performed for unrelated indications [5].

Thymic cysts are uncommon benign lesions of the anterior mediastinum, representing less than 5% of thymic masses. They may be congenital, arising from remnants of the thymopharyngeal duct, or acquired, particularly in inflammatory or degenerative settings. Most are discovered incidentally, as they remain asymptomatic in the majority of cases, with clinical manifestations mainly related to compressive effects when the lesion reaches a significant size [6].

The coexistence of a thymic cyst and a thymolipoma, in the absence of myasthenia gravis or associated autoimmune disease, may suggest either a coincidental association of two rare thymic lesions or a secondary transformation process, in which cystic changes develop within a pre-existing thymolipoma or, conversely, a thymic cyst. However, given the very limited number of reported cases, no definitive pathophysiological hypothesis can be established [4].

Although thymolipoma is a rare benign tumor of the thymus, its association with a thymic cyst is exceptionally uncommon. In our case, the lesion was incidentally detected on thoracic computed tomography and was initially suspected to represent a thymoma based on its mediastinal location and imaging characteristics. The final diagnosis was established only after histopathological examination of the surgical excision specimen, highlighting the diagnostic challenge posed by this unusual association.

From a clinical standpoint, both entities often share a silent or nonspecific mode of presentation, primarily related to a gradual compressive effect on adjacent mediastinal structures as the mass enlarges. The absence of systemic symptoms, persistent pain, or signs of local invasion favors a benign process, although these features alone do not allow malignant thymic tumors to be formally excluded [5].

Radiologically, distinguishing between a thymolipoma, a thymic cyst, or a mixed thymic lesion may be challenging. Imaging studies, particularly computed tomography and magnetic resonance imaging, can identify fatty and fluid components but are not always sufficient to establish a definitive diagnosis [7], especially in thymomas exhibiting cystic or degenerative changes [8]. Consequently, histopathological examination remains essential to confirm the benign nature of the lesions and to exclude a malignant thymic tumor or a cystic thymoma.

Histologically, thymolipoma is characterized by the presence of mature adipose tissue intimately admixed with normal thymic tissue, with preservation of lobular architecture [9], occasional Hassall's corpuscles, and, most importantly, the absence of cytological atypia, abnormal mitotic activity, or capsular invasion [10]. A thymic cyst is defined by a cystic cavity lined by a benign epithelium and associated with a fibrous wall that may contain residual thymic tissue, without atypical epithelial proliferation [7]. In contrast, malignant thymic tumors, particularly high-grade thymomas and thymic carcinomas, exhibit atypical epithelial proliferation, architectural disorganization, increased mitotic activity, and, above all, evidence of capsular or adjacent tissue invasion - features absent in the benign thymic cyst-thymolipoma association [9]. The differential diagnosis also includes thymic lymphoma, which is characterized by a diffuse, monoclonal lymphoid proliferation that effaces normal thymic architecture, without organized mature adipose tissue or a true epithelial-lined cystic wall [6].

Thus, the association between a thymic cyst and a thymolipoma should be recognized as a rare benign combination, provided that comprehensive histological analysis confirms the absence of malignant criteria. Complete surgical resection remains indicated for both diagnostic and therapeutic purposes, allowing definitive confirmation and ensuring an excellent prognosis, as recurrences are exceptional [5].

## Conclusions

Benign thymic lesions are uncommon and are often discovered incidentally because of their asymptomatic course. Thymic cysts and thymolipomas typically show an indolent evolution, but their imaging appearance may mimic malignant thymic tumors, creating a diagnostic challenge. Histopathological examination remains essential for confirming the benign nature of these lesions, while complete surgical excision provides both diagnosis and treatment with an excellent prognosis. The coexistence of a thymic cyst and a thymolipoma is exceptionally rare and highlights the importance of recognizing this association.
